# E-Liquids from Seven European Countries–Warnings Analysis and Freebase Nicotine Content

**DOI:** 10.3390/toxics10020051

**Published:** 2022-01-23

**Authors:** Patryk Krystian Bębenek, Vinit Gholap, Matthew Halquist, Andrzej Sobczak, Leon Kośmider

**Affiliations:** 1 Department of General and Inorganic Chemistry, Faculty of Pharmaceutical Sciences in Sosnowiec, Medical University of Silesia in Katowice, Jagiellonska 4, 41-200 Sosnowiec, Poland; patryk.krystian.bebenek@gmail.com (P.K.B.); asobczak@sum.edu.pl (A.S.); 2 Department of Pharmaceutics, School of Pharmacy, Virginia Commonwealth University, Richmond, VA 23298, USA; vinitgholap21@gmail.com (V.G.); halquistms@vcu.edu (M.H.)

**Keywords:** nicotine, nicotine form, e-liquids, European legislation

## Abstract

Electronic cigarettes are available in a variety of devices with e-liquids also available in many flavors, and nicotine concentrations, albeit less than 20 mg/mL in Europe. Given the dynamics of these products, it is important to evaluate product content, including labeling, nicotine content versus labeled claim, nicotine form, and other aspects that may help policy decisions and align with the Tobacco Product Directive (TPD). Herein, we performed a study on 86 e-liquids from seven European countries (Croatia, Czech Republic, France, Germany, Italy, Poland, and the United Kingdom) with 34 different liquid brands and 57 different flavors. Nicotine content versus labeled claim, labeling, volume, pH, and nicotine form (i.e., freebase nicotine) were evaluated. From all tested products, eight of them from Germany, Poland, and UK (from 3 to 18 mg/mL), met the ±2% criteria. The ±10% criteria was fulfilled by 50 (58.1%) liquids from all countries. Among 71 liquids which contained nicotine, (one e-liquid labeled as 6 mg/mL had no nicotine level quantified), the amount of freebase nicotine differed from 0 to 97.8%, with a mean value 56.5 ± 35.7. None of the tested liquids had nicotine salt listed in the ingredients. Therefore, a low level of freebase nicotine in some liquids was most likely achieved by added flavorings. All tested liquids presented in this study met the basic requirements of the TPD. There were differences in the scope of information about harmfulness, type of warnings on packaging, attaching leaflets, placing graphic symbols, and discrepancies between the declared and quantified nicotine concentrations.

## 1. Introduction

Electronic-cigarette companies have sold their products as a cheaper, tobacco-free, or smoke-free alternative to cigarettes, cigars, and other tobacco goods [1]. Marketing campaigns are focused on the attractiveness of these products: a variety of flavors, different designs, and devices perfect for tobacco smokers or people trying this type of product for the first time [2]. Companies presenting e-cigarettes focus on the absence of real tobacco in their devices and what comes with it—the lack of a characteristic irritating smell and ash—as a new way of a more socially acceptable form of nicotine consumption [3]. Although nicotine is an addictive component of tobacco, negative health effects are induced by other components of tobacco smoke [4,5]. E-cigarettes can be used with a wide range of nicotine concentrations, including without nicotine; however unlike traditional cigarettes, e-cigarettes do not contain tobacco and emit smoke, because their use is not based on combustion, which leads to lower harmfulness of e-cigarette aerosol [6]. There is little evidence that e-cigarette emissions harm the health of bystanders [6]. Using e-cigarettes can increase the amount of particulate matter in the air; however, the composition is different from that caused by cigarette smoke and the concentration is much lower, and sometimes at the same level as in rooms without smoking or using e-cigarettes [7,8,9,10,11].

The WHO Tobacco-Free initiative commissioned a report to help countries around the world develop policies to regulate e-cigarettes. This report, published in 2013, contained detailed political suggestions for countries regarding the regulation of e-cigarettes. These include: (1) a ban on the use of e-cigarettes wherever the use of traditional cigarettes is prohibited, (2) a ban on the sale of e-cigarettes to anyone who cannot legally buy cigarettes or other places where the sale of traditional cigarettes is prohibited, (3) apply the same marketing restrictions for e-cigarettes that apply to traditional cigarettes, (4) prohibition of using branded cigarettes or e-cigarettes, which promotes dual use, (5) a ban on the use of distinctive flavors in e-cigarettes like candy and alcohol flavors, (6) forbidding companies to make claims regarding the cessation of tobacco use (until e-cigarette manufacturers and companies provide sufficient evidence of this, that Electronic Nicotine Delivery System (ENDS) products can be effectively used to quit smoking) and (7) prohibiting e-cigarette companies from making health claims about their products, unless made by independent regulatory agencies, and (8) calls for standards to regulate the ingredients and functioning of the product [12,13].

The “Europe against cancer” program started in 1985, resulting in the introduction of a number of tobacco control measures and one of these was the 2001 Tobacco Products Directive (2001/37/EC), which regulates the production, sale and presentation of tobacco products [14,15,16]. In 2009, the European Commission published a report of this directive in the light of new market and scientific developments and the WHO Framework Convention on Tobacco Control (FCTC) [17]. The European Union (EU) Tobacco Products Directive was passed in 2014 and implemented in 2016. Article 20 of the Directive has brought forward specific regulations regarding components reporting, emissions, production quality control and potential design parameters that could reduce risk. At the same time, all members of the European Union banned placing on the market cigarettes containing characteristic flavors, such as menthol, chocolate, or vanilla since May 2020. However, these regulations do not apply e-cigarettes, which can be found with many different types of flavors. Among adolescent, flavors are especially appealing and increase youth preferences for e-cigarettes [18]. Flavored e-cigarettes also effect receptivity to use, willingness to use and perception on associated risk. Some studies present results that e-cigarettes can become a gateway for future cigarette use among youths [19,20]. The agents used in e-cigarettes to impart different flavors are widely recognized as not harmful when consumed in most consumer products available in the market. However, the potentially harmful effects on health during single inhalation and repeated inhalation of many of these flavoring agents are still barely known and uncertain [21]. The results of in vitro and laboratory studies indicate that fruit flavors, one of the most popular types of flavors added to e-cigarettes, have been associated with exposure to higher concentrations of known irritants agents during inhalation, lower activity of bronchial epithelial cells, and increased release of pro-inflammatory cytokines [22,23,24]. Fruit flavors are also implicated with the possibility of increasing the delivery of nicotine compared to other e-cigarette flavors, which may affect to the addictive potential of these products [25,26].

One of the greatest challenges surrounding e-cigarettes is whether these devices are used like recreational drugs like cigarettes or for abuse treatment. It is likely that e-cigarettes constitute both, making it difficult for regulatory efforts. The United Kingdom has long focused on the potential of using e-cigarettes as tools for tobacco harm reduction and smoking cessation. In 2015, Public Health England (PHE) published a report including information that e-cigarettes were approximately 95% safer than traditional smoking [27]. Furthermore, in 2010, the PHE created a possibility confirmed by English law for e-cigarettes as a medicine, what would involve meeting medicinal standards and advertising conditions for these products [27]. Taking into consideration the high costs of the application to get the license for e-cigarettes as medicine and the difficulty of meeting the medicinal requirements, since this report, no e-cigarette manufacturer has attempted to obtain a license. The UK Medicines and Healthcare Products Regulatory Agency set new rules in May 2016, introducing safety and quality standards for e-cigarettes, including restrictions on the total content of nicotine for all e-cigarette consumers, according to the European Union 2014 Tobacco Products Directive [28].

European countries like Denmark [29], Norway [30], Switzerland [31], and Sweden [32] that have registered e-cigarettes only for therapeutic purposes in the past have changed their law to dual-track regulations that permit them to be sold either as a consumer product, or medicine for therapeutical treatment. Some countries (Singapore, Thailand and Western Australia) completely banned the sale, and in special circumstances, the possession and use of all vaping products, even including those devices that did not contain nicotine [33].

In most European countries, e-cigarette regulation focuses on their classification as tobacco, and preparation for medicinal purposes or consumer products. Governments of some of these countries (e.g., Austria, Czech Republic, Denmark, Croatia, Ireland, Finland, Poland) established two or more classifications for e-cigarettes, which results in several regulatory approaches for these products [34]. Commonly used rules for classic tobacco products, like restriction to sale and advertisement, were included for e-cigarettes. Other tobacco control laws were expanded to e-cigarettes also, like e-cigarette-free public places and banning purchase laws for adolescents. About a third of countries that regulate e-cigarettes only apply existing tobacco control regulations to these products and fail to perform separate policies for e-cigarettes [34]. Some rules that have been adopted for tobacco products, such as health warning labels (HWLs), are challenges for e-cigarette manufacturers and legislation, considering that we currently have many different devices and different types of packaging. Furthermore, governments from European countries still have not decided on exactly what health warnings should be included on e-cigarettes and their packages, which results in different warnings used in the European Union, despite the Tobacco Product Directive. Few countries around the world tax e-cigarettes or liquids, and there were no policies about regulating the concentration of liquid ingredients, excluding nicotine levels [34].

The ambiguity in the regulatory approach in various EU countries was noted in the report of the European Commission [35]. The report concludes that Member States have had good experience with the implementation of some e-cigarette legislation, with the possibility for improvement in other specific areas. Pursuant to Art. 20 paragraph 2, more can be done to provide higher quality information, particularly toxicological data and uniform doses of nicotine during product consumption, such as by standardizing assessment methods.

In this survey, the team focused on which regulatory domains due to the Tobacco Directive were being applied to liquids, mainly on the warnings and HWLs on the liquid packaging. It was important to identify which information and HWLs are on the package and what they depend on. To achieve this goal, the team gathered information placed by manufacturers from the liquid package and bottle. The next step was to verify obtained information from liquids, compare them with regulations given by the Tobacco Directive, and collate data from samples with each other, including comparing nicotine level, HWLs on the package and bottle label, and other warnings included on labels. 

**Figure 1:**
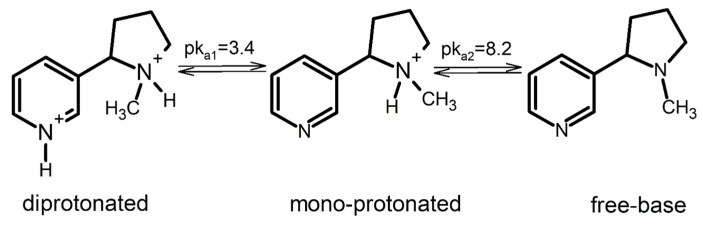
Nicotine forms.

Due to the latest data, the information related to the concentration of nicotine is especially important. The practice of producers to date was associated with the information about its total concentration. Meanwhile, nicotine depending on the pH can be presented as a freebase (non-protonated), mono-protonated, or diprotonated form (Figure 1). The freebase and protonated nicotine yield of the e-cigarettes is found to have different effects on the plasma nicotine concentration-time profile in vapers [36,37,38,39]. As the possible reasons for such differences are being studied, it becomes necessary to determine the freebase or protonated nicotine yield of liquids and classify them based on this yield. Such classification would eventually help in better regulation of the liquid/e-cigarette market [37,40].

## 2. Materials and Methods

In total, 86 liquids from seven European countries (Croatia, Czech Republic, France, Germany, Italy, Poland, and the United Kingdom) with 34 different liquid brands and 57 different flavors were used in this study. Randomly selected liquids were purchased at the turn of 2018 and 2019 in stationary stores (mainly kiosks, cigarette and tobacco stores or vape shops) in respective countries by researchers. The team obtained e-liquids with different nicotine concentrations; however, not every type of nicotine concentration was available in stationary stores. It is probably due to this fact that not every nicotine level is popular among users in respective countries. The nicotine level in individual liquids varies from 0 mg nicotine concentration to 18 mg per ml. The research group consisted of 14, 3, 23, 3, 15, 1, 14, 2 and 11 liquids with nicotine concentrations of 0 mg/mL, 1.5 mg/mL, 3 mg/mL, 4 mg/mL, 6 mg/mL, 9 mg/mL, 12 mg/mL, 16 mg/mL, and 18 mg/mL, respectively. All samples were stored in the refrigerator prior to analysis.

The e-liquids were grouped by country of purchase and type of flavor (fruity, sweet, menthol, tobacco groups). Flavor groups were assigned by two scientists based on labeling; in the case of one e-liquid where the results for classification differed, it was marked as “unassigned” (Energy Drink). Details of the e-liquid classification can be found in Supplementary Table S1.

The total nicotine in liquids was determined by a previously published method using the HPLC-PDA detection method [41]. All chromatographic conditions used were described previously [40]. A Waters Alliance 2695 quaternary pump HPLC equipped with a Waters 996 PDA Detector was used, along with a Hypersil Gold Phenyl column (150 mm × 4.6 mm, 3 µm, Thermo Scientific™, Greenville, NC, USA) and a Security Guard Cartridge Phenyl (4 mm × 2.0 mm, Phenomenex, Torrance, CA, USA). Waters Empower 2 software was used for processing data. 

Similarly, freebase nicotine was calculated using a 10× dilution approach followed by the Henderson Hasselbalch method using a TruLab pH 1310P (YSI Incorporated, Xylem Inc, Yellow Springs, OH, USA) potentiometric pH meter with a TruLine 15 glass electrode selective to H+ ions and containing silver chloride reference electrodes [40]. Limit of detection was 0.007 mg/mL and limit of quantification 0.02 mg/mL for e-liquid analysis.

Seventy-two refill solutions containing nicotine (in accordance to labeling) were analyzed further. The difference between labeled nicotine content and the quantified nicotine content were calculated. Data were analyzed using Statistica 13.0 software. Differences between the mean freebase nicotine content of refill solutions/declare nicotine content (for e-liquids with nicotine and labeled concentrations as 1.5, 3, 6, 12 and 18 mg/mL) or flavor (for sweet, fruity, menthol and tobacco) were examined using ANOVA, and Scheffe’s method was used for post hoc testing (*p* < 0.05).

## 3. Results

### 3.1. Health Warning Labels

On every tested liquid from countries that participated in this study which contained nicotine, manufacturers placed information about the nicotine concentration in mg/mL or in percentages. Twenty-seven (31.4%) of them had information about the total nicotine level per bottle and nicotine level per puff on their package. All information about nicotine concentration (nicotine level per ml, per bottle and per puff) had only 14 (16.3%) tested liquids. A total of 59 (68.6%) liquids also had carton packaging. A total of 28 (32.5%) tested liquids were bought without an additional box. All nicotine liquids had carton boxes (if it was included) with warnings about nicotine as a compound of the product. Forty-nine (57%) of them had warnings that it should not be used by children, adolescents, or those aged under 18. Eight (9.3%) tested liquids had warnings on their packaging in regard to pregnant women. General warnings like “attention” or “danger” were placed on 31 (36%) liquids. Information about toxicity properties of tested liquids were noticed on 15 (17.4%) samples. On 26 (30.2%), liquid producers placed more specific information about health risks linked with using this product, like “harmful if swallowed”, “wash hands thoroughly after handling”, “do not eat, drink or smoke when using this product”, “toxic to the skin”, “not allowed for people with cardiovascular diseases, high blood pressure and lung diseases”, and “not suitable for non-smokers”. Three (3.5%) liquids without nicotine had information about the presence of propylene glycol and its harmful effects on health. An acute toxicity symbol was on 30 (34.9%) of them. Danger or attention labels were placed on 35 (59.3%) boxes of tested liquids. Producers placed “not allowed under 18 HWLs on 25 (42.4%) samples; however, the “not allowed for pregnant” mark were set only on 14 (23.7%) of them. The “keep away from children” symbol was observed on 20 (33.9%) packages. In summary, from 59 liquids with an additional carton package, 47 (79.7%) of them had HWLs.

On four (4.6%) tested bottles, there were no warnings. Three (3.5%) of them contained nicotine and one did not. From 72 liquids with nicotine, only on 58 (80.5%) liquids, producers placed additional information about nicotine level or the presence of this alkaloid on the bottle label. On 65 (75.6%) of all tested samples, the team identified information about banning sale to or use by children, adolescents, or people under 18. Information like “not allowed for pregnant women” were placed only on 18 (20.9%) liquids. Warnings about danger or paying attention when using these products or about the general toxicity of these products were noticed on 33 (38.4%) samples. More specific information about toxic effects during the use of these products, like “toxic to the skin”, “harmful if swallowed”, “do not eat, drink or smoke when using this product”, “toxic to the skin”, “not allowed for people with cardiovascular diseases, high blood pressure and lung diseases” were set by manufacturers on 22 (25.6%) bottle labels. Only on two labels were there warnings about propylene glycol, and these liquids were without nicotine. In the case of other tested samples, information about the presence of propylene glycol in the ingredients section was observed, though without warnings directed to consumers. Nineteen (22.1%) liquids from the 86 tested in this study had no HWLs on the bottle labels. Only four (4.6%) of them were without nicotine. Sixteen (18.6%) liquids which contained nicotine with different levels of this alkaloid and no HWLs about toxicity or paying attention were noticed in this survey. On 58 (67.4) bottle labels, we identified HWLs like “attention”, “danger”, or “acute toxic”. HWLs like “not allowed for children” or “not allowed under 18” were placed by producers on 45 (52.3%) labels on bottles. Information conducted with health effects for pregnant women were noticed on 19 (22.1%) liquid bottles.

Forty (46.5%) tested samples had additional information about the liquid’s components, how it should be used, and even more descriptions of the side effects of these liquids. From 86 liquids, the basic components of 83 (96.5%) of them were glycerin and propylene glycol. In three (3.5%) liquids, the main components were propane-1,2,3-triol and propane-1,2-diol. In one (1.2%) liquid, the main component of the liquid base was propane-1,2-diol. Liquids with different flavors had additional substances, like geraniol, vanillin, methyl cinnamate, or d-limonene, that have a characteristic smell and taste when used. All nicotine-containing and non-nicotine containing refill containers in this study were child- and tamper-proof, with protection against breakage and leakage.

#### 3.1.1. Germany

Liquids from Germany fulfilled requirements presented in the European Tobacco Directive. The product packaging had appropriate health warnings and a list of ingredients. Manufacturers indicated a nicotine concentration per ml on each label; however, information about total nicotine content was only on four of them, and did not consider the delivery dose. The nicotine level of all tested liquids from Germany was less than or equal to 20 mg/mL, and the volume of their refill bottles did not exceed 10 mL. Liquids in this study did not contain other addictive substances, except for nicotine. Health warnings like “this product contains nicotine which is a highly addictive substance” or “the product must be kept out of reach of children” or symbols indicating toxicity or danger appeared on the package or on the bottle label.

#### 3.1.2. United Kingdom

From 24 tested liquids from the UK, only eight (33.3%) of them placed information about the total nicotine concentration on the package or bottle. We observed the nicotine concentration per dosage only on seven (29.2%) liquids from the UK, which participated in this survey. The nicotine level of all tested liquids from the UK, like in liquids from Germany, were less than or equal to 20 mg/mL, and the volume of their refill bottles did not exceed 10 mL. Liquids in this study did not contain other addictive substances, except for nicotine. Health warnings were present on all tested liquids, even on products without nicotine. Information placed on liquids by producers mostly concerned things like keeping it out of the reach of children, not allowing it for pregnant women, and information about the concentration of nicotine and the addictive properties of this substance. On liquids which did not contain nicotine, producers placed information about propylene glycol. All tested products from the UK have symbols about possible risks, toxicity, and danger after usage.

#### 3.1.3. Poland

From 28 liquids with nicotine from Poland, on 10 (35.7%) of them, information about total nicotine amount was present; however, on 16 (57.1%) of them, information about nicotine level per dosage was found. The nicotine level of all tested liquids from Poland was less than or equal to 20 mg/mL, and the volume of their refill bottles did not exceed 10 mL. Except nicotine, there were no other addictive substances in the liquid components listed. Eight (28.6%) of all participating liquids in this study had textual information about keeping it out of the reach of children; however, these products had marks which symbolized it being banned for adolescents. On 8 (22.8%) of 35 tested liquids from Poland, manufacturers did not place symbols about toxicity and banned usage for children.

#### 3.1.4. Croatia

On every liquid from Croatia, the manufacturer placed information about the nicotine concentration per ml and per dosage; however, they did not include the total nicotine level per bottle. The nicotine concentration in refill bottles of all tested liquids from Croatia was less than or equal to 20 mg/mL, and their volume did not exceed 10 mL. Nicotine was the only addictive substance which was included in the compounds section on the label. All tested liquids with nicotine from Croatia had information or symbols about toxicity, danger, harmful effects after ingestion, or being banned for adolescents. The team observed a notification about the product’s harmful effects on pregnant women only on three of them.

#### 3.1.5. Czech Republic

On all liquids from the Czech Republic which participated in this study, producers placed information about the nicotine concentration per mL. On three of them, manufacturers placed information about the total nicotine amount per bottle, and the team observed information about the nicotine level per dosage on none of them. All liquids from the Czech Republic had a nicotine concentration lower than or equal to 20 mg/mL, and the volume of refill containers did not exceed 10 mL. Nicotine was the only addictive substance placed by manufacturers on the label. All tested products from the Czech Republic had symbols about the possible risks, toxicity, and danger after usage; however, three of them had information and symbols only on the external carton package. On every package, information about it being banned for children and pregnant women was present.

#### 3.1.6. Italy and France

Liquids from Italy had information about the nicotine concentration per ml and total nicotine amount per bottle, except for the nicotine level per dosage. Their refill containers did not exceed 10 mL, and their nicotine concentration was lower than 20 mg/mL. Producers placed information and symbols about toxicity, danger, and the product being banned for children and adolescents. The same information was observed on labels on liquids from France; however, the manufacturers did not place notifications about the total nicotine amount per bottle, and as in Italy, there were no symbols or information about the product being banned for pregnant women.

All tested liquids presented in this survey fulfilled the requirements presented in the European Tobacco Directive; however, the team observed differences between the tested samples. The differences in most cases depended on the liquid manufacturer, not the origin of the liquid. Some producers placed additional information, like the total nicotine amount or nicotine level per dosage, but there were no requirements about these parameters in the directive or local regulations. The divergences observed mainly concerned the type of symbols and their meanings. On some labels, the producers only placed symbols about danger, and on others, they included pictograms related to toxicity. Some manufacturers put information or symbols about its harmful effects on pregnant women, and others did not. In some cases, the team noticed very specific and accurate information about health risks connected with the liquid’s usage; however, in most of the samples, there were only general notifications about its effects on user health. Some liquids without nicotine had information about risks and health effects if swallowed, or information about it not being allowed for pregnant women and children, while others only had information about keeping away from adolescents. The main purpose of the European Tobacco Directive was to unify regulations concerning liquids in European countries; however, these guidelines are still not precise and provide the possibility to obtain liquids with the same nicotine concentration, but with different health warning symbols and textual warnings.

Because liquids do not have standardized guidelines, we used the USP and ICH guidelines for the liquid analysis with the acceptance criteria of ±2% for and ±10%, as followed by pharmaceutical manufacturers for labeling claims. From all tested products, only two of them which were manufactured in China and available in Poland met the ±2% criteria. The ±10% criteria was fulfilled by liquids from Italy and Czech Republic. In two nicotine-free liquids, nicotine was present—one from Poland with 0.02 mg/mL nicotine content, and the second one from Croatia, with 0.05 mg/mL nicotine concentration. Sixty-two of the tested products had a higher deviation than ±2%. Thirty-nine of them were with a nicotine concentration between >0 to 6 mg/mL (Group I). A total of 11 liquids which exceeded a ±2% deviation range were from a group with a nicotine level from 9 to 12 mg/mL (Group II). In the group with the highest nicotine range between 16 and 18 mg/mL (Group III), 12 marked liquids had not met the ±2% criteria. In the ±10% deviation, the lowest nicotine concentration group (Group I) had the highest number of exceeded samples. In the second group (Group II), from 15 tested liquids, three of them failed to meet the ±10% criteria. In the last group (Group III), none of those which participated in this study exceeded the 10% range. From all tested liquids in this survey, six liquids had a nicotine concentration higher than the labeled claim. Three of them were in the group with the lowest nicotine level. One of the exceeded samples was part of a group with a nicotine concentration between 9 and 12 mg/mL (Group II). In the last group (Group III), only two liquids had higher nicotine levels than declared by the manufacturers on the label. The lowest nicotine concentration was investigated in 56 of all liquids which participated in this study. Thirty-six of them were a part of the lowest nicotine content group (Group I). In the second and third groups (Group II and III), there were 10 samples with a lower nicotine level compared to the label.

In samples obtained from Italy, both tested liquids had a different nicotine concentration than the content presented on the label. One with 9 mg/mL nicotine had lower nicotine content by 13.2%. In the second one, the nicotine level was higher by 3%. Italian liquids failed to meet the ±2% criteria, and the liquid labeled as 18 mg/mL passed the ±10% criteria. All samples from France had a lower concentration of nicotine (by 15.4 ± 3.9%, *n* = 3) compared to the content declared by the producers on the packaging (4 mg/mL). Samples from this country did not meet the criteria of ±2% and the criteria of ± 10%. Three marked liquids from Germany had a higher nicotine level by (1.6 ± 2.21%, *n* = 3). For five German samples, the team found there was a lower nicotine level than that declared (8.3 ± 6.5%, *n* = 5). Five liquids from Germany failed the ±2% criteria, and for one, the ±10%. All samples from the Czech Republic had a lower nicotine concentration than that declared on average by 4.1 ± 1.9%; *n* = 6. All liquids from this country failed to meet the 2% criteria; however, all of them passed the 10% criteria. In the case of liquids from Croatia, six of them exceeded the ±2% limit, and one of them also did not meet the ±10% limit. All marked samples had a lower nicotine concentration than the declared value (mean 8.6 ± 5.8%, *n* = 6). For Polish liquids, 23 (65.7%) of 35 samples had a lower nicotine concentration than the content on the labels by (14.6 ± 19.9%, *n* = 23). Five liquids had a higher nicotine level in comparison to the value on the package (4.5 ± 3.8%, *n* = 5). The 2% criteria was unacceptable in 24 (68.6%) liquids, and 9 (25.7%) did not pass the 10% criteria. Samples from the United Kingdom revealed a lower nicotine concentration than the level presented by producers on labels (11.6 ± 11.7%, *n* = 17). Two of all liquids from this country had higher nicotine content compared to the concentration placed on the package (higher by 142.09% and 0.083%). The criteria of ±2% and ±10% did not pass 16 and 7 samples, respectively.

### 3.2. Nicotine Content

The comparison of labeled and calculated nicotine concentration was performed for all 86 e-liquids. Fourteen chosen liquids from four countries had a nicotine concentration labeled as 0 mg/mL. Twelve had no detectable nicotine level, and the remaining two liquids had a determined nicotine level of 0.02 and 0.05 mg/mL from Poland and Croatia, respectively. From all tested products, eight of them from Germany, Poland, and the UK (from 3 to 18 mg/mL) met the ±2% criteria. The ±10% criteria fulfilled 50 (58.1%) liquids from all countries excluding France, where only two liquids were tested, with a quantified concentration lower by 14.3% and 19.9% (both labeled as 4 mg/mL). Only one liquid had a concentration higher than that claimed by more than 10%, where the quantified concentration for this liquid was 3.63 and labeled 1.5 mg/mL. Twenty-one liquids had a concentration lower by more than 10%, with one liquid with a labeled nicotine concentration of 6 mg/mL with no traces of nicotine in it (liquid from Poland). The mean difference of quantified nicotine versus the label for 72 liquids, which had a labeled nicotine level of 1.5 or higher, was −7.5 ± 22.7%. There was no statistical difference in the relative difference between countries or labeled nicotine (*p* > 0.05), probably due to the small sample amounts of 1.5 mg/mL and 16 mg/mL. In Table 1 and Table 2, the mean values for relative differences are presented in relation to labeled nicotine and country of origin. In Supplementary Table S2, we present the results for non-nicotine e-liquids, as those were not statistically analyzed. 

### 3.3. Freebase Nicotine Content

Among 71 liquids which contain nicotine (one e-liquid labeled as 6 mg/mL had no nicotine level quantified), the amount of freebase nicotine differed from 0 to 97.8%, with a mean value of 56.5 ± 35.7. None of the tested liquids contained nicotine salt, so a low level of freebase nicotine in some liquids was achieved probably by added flavorings. Fifty percent of tested liquids had a freebase nicotine level higher than 74.4%, 25% lower than 17.2%, or higher than 86.7%.

**Figure 2:**
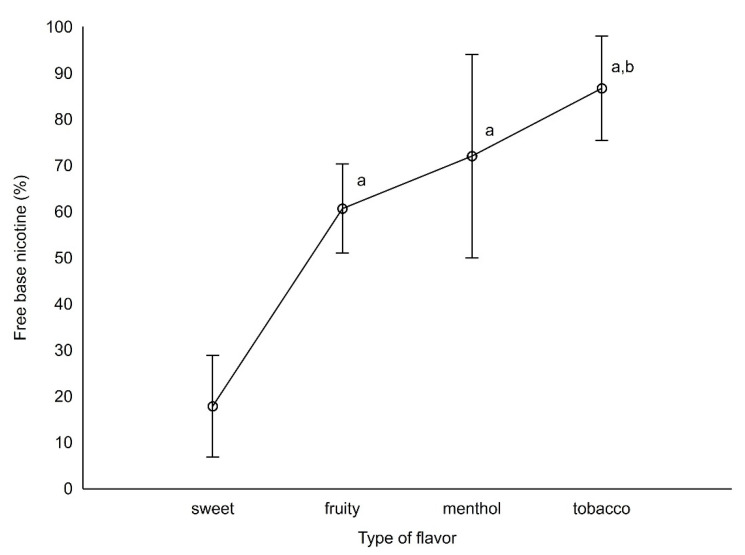
Differences in freebase nicotine content between different types of flavors. Liquids which differ statistically from sweet or fruity liquids were marked as a and b, respectively (*p* < 0.05, Scheffe test).

**Figure 3:**
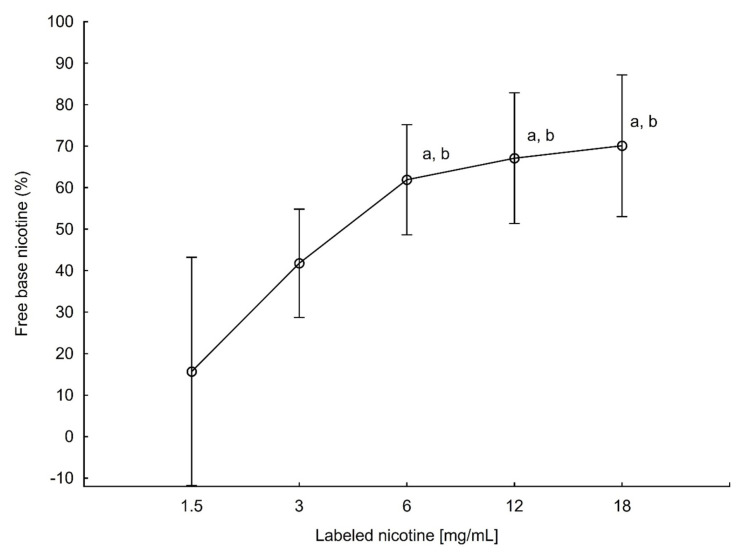
Differences in freebase nicotine content between different labeled nicotine concentrations. Liquids which differed statistically from 1.5 mg/mL nicotine liquids or 3 mg/mL liquids were marked as a and b, respectively (*p* < 0.05, Scheffe test).

Liquids from France and Italy (as only liquids with 4 and 9 mg/mL), as well as 16 mg/mL e-liquids were excluded from statistical analysis for association of nicotine content on the freebase nicotine level due to a small sample size. Detailed freebase nicotine ratios broken into countries or labeled nicotine are presented in Table 1 and Table 2. There were no statistical differences between countries in freebase nicotine content (*p* > 0.05), in contrast to types of flavor and labeled nicotine (*p* < 0.001 and *p* = 0.0012, respectively). Details can be found in Figure 2 and Figure 3 and Table 1 and Table 2. Sweet types of liquids differed statistically from fruity, menthol, and tobacco flavors; fruity liquids differed from tobacco-type liquids in freebase nicotine content (both *p* < 0.05). There was no statistical difference in relation to freebase nicotine between countries and concentrations > 0.05. In Table 3, detailed results for freebase nicotine content in different flavoring groups can be found. 

## 4. Discussion

All tested liquids presented in this study were generally compliant with the European Tobacco Directive. However, the team observed differences between the samples tested in some areas. In most cases, these differences depended on the producer of the liquid, not the origin of the liquid. Some manufacturers provided additional information, such as the total nicotine level per dose, although there are no requirements for these parameters in the directive or local legislation. The observed discrepancies mainly regarded the type of symbols and their marks. Some labels had only symbols of danger, while others had pictograms related to toxicity. Some producers included information or symbols of the product’s harmful effects on pregnant women, while others did not. In some cases, very detailed and accurate information about the health risks of using the liquids were present; however, only general notifications about the health effects of users appeared in most samples. Some of the nicotine-free liquids contained information about risks and health effects if swallowed, or prohibition of use for pregnant women and children, while others merely contained information about keeping away from adolescents. The main goal of the European Tobacco Directive was to harmonize the regulations on liquids in European countries, but for the time being, these guidelines are still not precise and allow consumers to purchase liquids with the same nicotine concentration, but with different warning symbols and text warnings.

In our study, all tested packages of liquids contained information about the nicotine content in mg/mL (100%) if nicotine was present. Approximately 31% of them also contained information about the amount of nicotine delivered per dose; however, the information about the content in mg/mL and per dose was present in only 16.3% of tested liquids. In the outer packaging, warning information about the nicotine content was observed on each of the tested samples, while the percentage of warning symbols on the outer packaging constituted 79.6%. On bottle labels of 80.5% of the tested liquids, information was present about the concentration of nicotine, and on 77.9%, there were warning symbols. There was no warning text (4.6%) on the labels of the four bottles. The percentage of leaflets in the case of the tested samples was 46.5%.

Our observations regarding the discrepancy in the characteristics of liquids were consistent with the results obtained by other authors. Girvalaki et al. [42] observed that after the introduction of the European Tobacco Directive, the compliance of the volume of liquid refilling bottles (≤10 mL in vials) increased from 86.9% to 94.4%, *p* = 0.008. They also observed compliance with the maximum levels of nicotine concentration (100.0%) in the tested samples, while the percentage of products reporting nicotine delivery per dose increased from 0.9% to 43.9%, *p* < 0.001. The percentage of products containing a package leaflet also increased from 26.2% to 53.3%, *p* < 0.001. Additionally, the number of warnings on a bottle, box, or leaflet increased significantly after the introduction of the Directive. The presence of textual warnings on the box increased from 2.8% to 72.0%, *p* < 0.001, on bottles from 19.6% to 32.7%, *p* = 0.022, and on the leaflet from 13.1% to 42.1%, *p* < 0.001. Eighty-six percent of the tested products had some form of warnings in the period after the introduction of the directive, compared with 32.7% of products before the implementation of the directive (*p* < 0.001).

Very important information for the user is the amount of nicotine concentration placed on the package. This is due to the fact that the actual lower concentration of nicotine by the concentration declared by the manufacturer may, for some e-cigarette users, be associated with a compensatory effect of deeper and more frequent puffs. It is related to inhalation of a larger number of toxic compounds that may be degradation products of the liquid. As a result, a higher nicotine concentration in the liquid than the declared value may increase the potential of nicotine addiction.

Our research shows that among all tested liquids, in 7% of them, the marked nicotine concentrations were higher than the declared content by the manufacturer. Lower content was determined in the case of 77.8% of liquids. The difference between the quantified nicotine content and the manufacturer’s was declared for 72 liquids, where a nicotine level described on the packaging of 1.5 mg/mL or higher was −7.5 ± 22.7%.

Our observations about the differentiation of nicotine concentration provided by the manufacturer and its actual concentration confirmed the previous results obtained by other research teams. A retrospective analysis of 23 studies from 2013–2020 showed that out of 545 liquids, 107 contained nicotine at a level above 20 mg/mL. Importantly, many of these liquids came from the USA, where there is no legal upper limit of nicotine concentration in liquids. Only 15 liquids in this group came from countries with a nicotine limit of 20 mg/mL (Great Britain, Greece, France, and Poland). The most common case of mislabeling was 0% to 5% of the nicotine concentration stated by the manufacturer. The second largest frequency of mislabeling was in the range of 10–20% [43].

Over the past few years, e-liquids have started to be advertised as liquids containing nicotine salts. This approach not only masks the irritating taste of nicotine, but can also affect the intensity of nicotine absorption into the bloodstream. This is due to the form in which it is absorbed into the body.

According to Pankow’s theory, nicotine in aerosols occurs in the form of a freebase or in protonated form (salt), depending on the chemical composition of the aerosol, which, in the case of e-cigarettes, is closely related to the composition of the liquid. Freebase, due to its volatility, occurs mainly in the gaseous state, while protonated nicotine occurs mainly in the form of solid particles (droplets) [44]. Recently, David et al. experimentally proved that protonated nicotine remains in the aerosol droplets by use of ion-trapping and the Raman scattering technique [45]. Therefore, nicotine has a better chance of reaching the lungs where it dissociates, and the freebase form is absorbed by alveolar cells [46,47]. The volatility of free nicotine and its gaseous presence means that it is more likely to remain in the upper respiratory tract; hence, the absorption of nicotine is slower than that from the lungs [48]. Additionally, there is a greater chance of exhaling the gas fraction containing nicotine as a freebase [39,49]. In summary, the amount of nicotine reaching the lungs influences the plasma concentration of nicotine, and the amount of nicotine reaching the lungs is influenced by the form of inhaled nicotine. Consequently, two liquids with the same total nicotine concentration but with a different form of nicotine (free versus salt) can potentially cause significant variations in plasma nicotine levels. We would observe a higher concentration of nicotine in the plasma with a large amount of protonated form.

The observed differences in the pH of liquids for different nicotine concentrations declared by the manufacturer (range 5.38 ± 1.10 ÷ 9.04 ± 0.43) were the basis for a hypothesis about the effect on the pH of flavorings added to liquids. Consequently, in the tested liquids, nicotine occurred in both discussed forms, but in a different quantitative ratio. The smallest amount of free nicotine was found in sweet liquids and increased in the following order: fruit, menthol, tobacco. 

This work has some limitations. First is the method for quantifying the free nicotine base in e-cigarettes. It was limited by factors such as the arbitrary dilution factor and the unknown H ‘activity factor due to unknown ion concentrations. However, we believe that the impact of these restrictions is negligible [39,40]. Secondly, the division into flavor groups was based on the description on the packaging. In approximately 12, the description did not allow assignment of the liquid to the appropriate group. Finally, experienced vapers were included whom, after using it, assigned the liquid to one of four groups; however, this could be considered subjective.

To fully confirm our hypothesis, clinical trials are needed to determine the level of nicotine in the plasma of vapers using liquids with the same starting concentration but a different ratio of nicotine freebase and protonated nicotine (different flavors). Additionally, it is important to understand the absorption profile of both forms of nicotine under different vaping conditions [50]. Recently, Gholap et al. described various factors that can affect the freebase/protonated nicotine yields from the e-cigarettes in detail. Such research acts an important stepping-stone towards understanding the absorption profiles of nicotine under various user conditions. Therefore, future research should be conducted considering a multidimensional approach to aid in better regulation of e-cigarettes.

## 5. Conclusions

All tested liquids presented in this study met the basic requirements of TPD. There were differences in the scope of information about harmfulness, the type of warning on the packaging, attaching leaflets, the placement of graphic symbols, and discrepancies between the declared nicotine concentrations and its actual concentration. An important aspect of this work is the demonstration that flavoring substances were associated with different ratios of the form of nicotine, which may have an impact on inhaled nicotine form and plasma nicotine levels and hence, on addiction, which requires further research. We believe this aspect of the work is important in the context policy and practice in the field of tobacco control, especially as the use of nicotine salts and modified wicks has been found to be associated with higher rates of addiction [51].

## Figures and Tables

**Table 1:** Mean difference in nicotine content divided into countries in e-liquids with labeled nicotine level above 0 mg/mL.

Country	N	Relative Difference (%) Mean ± SD	Freebase Nicotine (%) Mean ± SD	pH Mean ± SD
Croatia	6	−8.65 ± 5.84	40.4 ± 40.1	7.65 ± 1.67
Czech Republic	6	−4.38 ± 1.42	81.8 ± 9.4	8.63 ± 0.26
France	3	−15.42 ± 3.88	4.2 ± 3.4	6.51 ± 0.83
Germany	8	−4.53 ± 7.23	88.8 ± 5.5	9.12 ± 0.25
Italy	2	−5.11 ± 11.47	72.5 ± 20.1	8.65 ± 0.47
Poland	28	−11.23 ± 19.40	61.9 ± 30.0 *	8.26 ± 1.16
United Kingdom	19	−2.88 ± 36.89	38.9 ± 39.2	7.46 ± 1.40

Note: * *n* = 27 due to one e-liquid with nicotine undetected.

**Table 2:** Mean difference in nicotine content of different nicotine labeled refill solutions and freebase nicotine content in e-liquids with labeled nicotine level above 0 mg/mL.

Labeled Nicotine Concentration (mg/mL)	N	Relative Difference (%) Mean ± SD	Freebase Nicotine (%) Mean ± SD	pH Mean ± SD
1.5	3	30.27 ± 96.91	0.5 ± 0.4	5.38 ± 1.10
3	23	−10.40 ± 11.57	35.0 ± 34.9	7.38 ± 1.35
4	3	−15.42 ± 3.88	4.2 ± 3.4	6.51 ± 0.83
6	15	−12.96 ± 24.66	67.6 ± 28.1 *	8.50 ± 0.70
9	1	−13.22	58.3	8.32
12	14	−5.96 ± 7.41	77.9 ± 16.0	8.83 ± 0.44
16	2	−5.59 ± 0.66	81.8 ± 5.7	8.83 ± 0.17
18	11	−3.92 ± 4.38	84.9 ± 15.3	9.04 ± 0.43

Note: * *n* = 14 for mean and SD analysis due to one e-liquid with undetected nicotine.

**Table 3:** Mean freebase nicotine content of different nicotine labeled refill solutions.

Type of Flavor	Number of E-Liquids in a Group	Number of E-Liquids with Nicotine	Freebase Nicotine (%) Mean ± SD *	pH Mean ± SD *
Fruit	34	26	60.7 ± 27.9%	8.22 ± 1.18
Sweet	22	20	17.9 ± 30.8%	6.83 ± 1.23
Tobacco	24	19	86.8 ± 7.5%	9.05 ± 0.31
Menthol	5	5	72.1 ± 20.9%	8.67 ± 0.47
Unassigned	1	1	66.7%	8.47

Note: * Calculated only for e-liquids containing nicotine.

## Data Availability

The datasets generated during and/or analyzed during the current study are available from the corresponding author on reasonable request.

## Supplementary Materials

The following are available online at https://www.mdpi.com/article/10.3390/toxics10020051/s1, Table S1: Detailed e-liquid data, Table S2: Results for e-liquids with nicotine level labeled as 0 or with no quantified nicotine level.
