# Supplementary material for: E-Liquids from Seven European Countries–Warnings Analysis and Freebase Nicotine Content
Source: Toxics. 2022 Jan 23;10(2):51. doi: 10.3390/toxics10020051 (PMC8875468; doi:10.3390/toxics10020051)
Supplement: Supplementary file 1 [file toxics-10-00051-s001.zip › toxics-1518492-supplementary.pdf]

toxics MDPI

Supplementary Materials: E-liquids from Seven European Countries–Warnings Analysis and Freebase Nicotine Content

Patryk Krystian Bębenek, Vinit Gholap, Matthew Halquist, Andrzej Sobczak and Leon Kośmider

Table S1. Detailed e-liquid data.

Sample code	Country	Nicotine [mg/ml]	Type of flavor	Flavor	Warnings on the package	Marks on the package	Warnings on the bottle label	Marks on the bottle label
P2	Poland	18	fruity	Raspberry	Nicotine	Acutely toxic, Not allowed under 18	No information	No information
P6	Poland	18	menthol	Green mint	Nicotine, Keep away from children, Not allowed for pregnant women	Attention, Not allowed under 18, Not allowed for pregnant women	Nicotine contain	Warning nicotine contain
P7	Poland	18	fruity	Pineapple	Danger, Acute toxic, Nicotine, Not allowed under 18	Dangerous, Acutely toxic, Not allowed under 18	Dangerous, Nicotine contain, Not allowed under 18	Dangerous, Acutely toxic, Not allowed under 18
P16	Poland	18	tobacco	Desert ship	Danger, Acute toxic, Nicotine (without information in black-white frame), Keep away from children	Danger, Acute toxic	Danger, Acute toxic, Keep away from children, Nicotine contain	Danger, Acute toxic
C5	Croatia	18	tobacco	USA Mix	Acute toxic, Danger, Nicotine contain, Toxic after ingestion, Keep away from children	Acute toxic, Danger, Not allowed under 18, Keep away from children, Not allowed for pregnant, Not for consumption	Acute toxic, Danger, Nicotine, Toxic after ingestion, Not for consumption, Keep away from children	Acute toxic, Danger, Not allowed under 18, Keep away from children, Not allowed for pregnant, Not for consumption
G1	Germany	18	tobacco	Tobacco RY4 Blend	Nicotine contain, Not allowed for pregnant, Keep away from children, Not allowed under 18, Not allowed for people with cardiovascular diseases, high blood pressure and lung diseases, not suitable for non-smokers	Not allowed under 18, Not allowed for pregnant	Nicotine contain, Not allowed for pregnant, Keep away from children, Not allowed under 18, Not allowed for people with cardiovascular diseases, high blood pressure and lung diseases, not suitable for non-smokers	No information
G8	Germany	18	tobacco	Golden Blend	Nicotine contain, Keep away from children, Danger	Danger, Acute toxic	Nicotine, Keep away from children, Not allowed for people with	Attention, Acute toxic

							cardiovascular diseases and allergies	
P25	Poland	18	fruity	Apple	Danger, Nicotine contain, Toxic to the skin, Keep away from children	No information	Danger, Acute toxic, Not allowed under 18	No information
I2	Italy	18	tobacco	Classic	Nicotine contain, Keep away from children, Attention	Not allowed under 18, Attention, Acute toxic	Nicotine contain, Keep away from children, Attention	Acute toxic, Attention
UK18	United Kingdom	18	fruity	Watermelon	Contains nicotine. Toxic if swallowed. Wash hands thoroughly after handling. Do not eat, drink or smoke when using this product. Keep out of reach of children	Danger, Acute toxic, Keep away from children, Nicotine contain	Contains Nicotine	Danger, Acute toxic, Keep away from children, Nicotine contain
UK23	United Kingdom	18	tobacco	Tobacco	Contains nicotine. Toxic if swallowed. Wash hands thoroughly after handling. Do not eat, drink or smoke when using this product. Keep out of reach of children	Danger, Acute toxic, Keep away from children, Nicotine contain	Contains Nicotine	Danger, Acute toxic, Keep away from children, Nicotine contain
CZ4	Czech Republic	16	fruity	Apple	Nicotine contain, Attention, Harmful if swallowed	Danger, Acute toxic, Keep away from children, Nicotine contain	Attention, Acute toxic, Not allowed under 18, Not allowed for pregnant	Danger, Acute toxic, Keep away from children, Nicotine contain
CZ6	Czech Republic	16	sweet	Blueberry Cream	Nicotine contain, Attention, Harmful if swallowed	Danger, Acute toxic, Keep away from children, Nicotine contain	Attention, Acute toxic, Not allowed under 18, Not allowed for pregnant	Danger, Acute toxic, Keep away from children, Nicotine contain
P5	Poland	12	fruity	Orange	Nicotine, Keep away from children, Not allowed for pregnant women	Attention, Not allowed under 18, Not allowed for pregnant	Nicotine contain	Warning nicotine contain
P8	Poland	12	fruity	Kiwi	Attention, Acute toxic, Nicotine, Not allowed under 18	Attention, Not allowed under 18	Attention, Nicotine contain, Not allowed under 18	Attention, Not allowed under 18
P9	Poland	12	fruity	Raspberry-Orange	Danger, Acute toxic, Nicotine, Keep away from children	Danger, Acute toxic, Not allowed under 18, Not allowed for pregnant	Danger, Acute toxic, Keep away from children, Nicotine contain	Danger, Acute toxic, Not allowed under 18, Keep away from children, Not allowed for pregnant

C4	Croatia	12	tobacco	Tobacco	Acute toxic, Attention, Nicotine contain, Keep away from children, Not for consumption	Acute toxic, Attention, Not allowed under 18, Keep away from children, Not allowed for pregnant, Not for consumption	Acute toxic, Attention, Nicotine, Harmful after ingestion, Not for consumption, Keep away from children	Acute toxic, Attention, Not allowed under 18, Keep away from children, Not allowed for pregnant, Not for consumption
G3	Germany	12	tobacco	American Blend Gold	Nicotine contain, Not allowed for pregnant, Keep away from children, Not allowed under 18, Not allowed for people with cardiovascular diseases, high blood pressure and lung diseases, not suitable for non-smokers	Not allowed under 18, Not allowed for pregnant	Nicotine contain, Not allowed for pregnant, Keep away from children, Not allowed under 18, Not allowed for people with cardiovascular diseases, high blood pressure and lung diseases, not suitable for non-smokers	Acute toxic, Attention, Not allowed under 18, Not allowed for pregnant
G6	Germany	12	tobacco	Golden Blend	Attention, Nicotine contain, Keep away from children	Attention, Acute toxic,	Attention, Nicotine contain	Attention, Acute toxic
P18	Poland	12	fruity	Cherry	No box	No box	Attention, Nicotine contain, Keep away from children, Harmful if swallowed	Attention, Acute toxic,
P30	Poland	12	fruity	Forest fruits	Nicotine contain, Attention, Keep away from children	No information	Attention, Acute toxic, Keep away from children, Not allowed under 18, Not allowed for pregnant	No information
P31	Poland	12	tobacco	Tobacco	Nicotine contain, Attention, Keep away from children, Toxic to the skin	No information	Danger, Acute toxic, Not allowed under 18,	No information
CZ2	Czech Republic	12	fruity	Strawberry	Nicotine contain, Attention, Harmful if swallowed	No information	Attention, Acute toxic, Not allowed under 18, Not allowed for pregnant	No information
CZ3	Czech Republic	12	menthol	Ice Vape	Nicotine contain, Attention, Harmful if swallowed	No information	Attention, Acute toxic, Not allowed under 18, Not allowed for pregnant	No information
UK17	United Kingdom	12	fruity	Watermelon	Contains nicotine. Toxic if swallowed. Wash hands thoroughly after handling. Do not eat,	Danger, Acute toxic, Keep away from children, Nicotine contain	Contains Nicotine	Danger, Acute toxic, Keep away from children,

					drink or smoke when using this product. Keep out of reach of children			Nicotine contain
UK21	United Kingdom	12	tobacco	Tobacco	Contains nicotine. Toxic if swallowed. Wash hands thoroughly after handling. Do not eat, drink or smoke when using this product. Keep out of reach of children	Danger, Acute toxic, Keep away from children, Nicotine contain	Contains Nicotine	Danger, Acute toxic, Keep away from children, Nicotine contain
P32	Poland	12	sweet	Vanilla Custard Cream	Contains nicotine. Toxic if swallowed. Wash hands thoroughly after handling. Do not eat, drink or smoke when using this product. Keep out of reach of children	Danger, Acute toxic, Keep away from children, Nicotine contain	Contains Nicotine	Danger, Acute toxic, Keep away from children, Nicotine contain
I1	Italy	9	menthol	Menthol	Nicotine contain, Keep away from children	Not allowed under 18, Attention, Acute toxic	Nicotine contain, Keep away from children, Attention	Acute toxic, Attention
P1	Poland	6	menthol	Mojito	Nicotine	Acutely toxic, Not allowed under 18	No information	No information
P3	Poland	6	sweet	Black currant	Nicotine	Acutely toxic, Not allowed under 18	No information	No information
P4	Poland	6	tobacco	Montana tobacco	Nicotine	Attention, Not allowed under 18, Not allowed for pregnant	Nicotine contain	Warning nicotine contain
C2	Croatia	6	fruity	Bluberry Mint	Acute toxic, Attention, Nicotine contain, Not allowed under 18, Keep away from children	Acute toxic, Attention	Acute toxic, Attention, Nicotine contain, Not allowed under 18, Keep away from children	Acute toxic, Attention
G2	Germany	6	tobacco	American Blend Gold	Nicotine contain, Not allowed for pregnant, Keep away from children, Not allowed under 18, Not allowed for people with cardiovascular diseases, high blood pressure and lung diseases, not suitable for non-smokers	Not allowed under 18, Not allowed for pregnant	Nicotine contain, Not allowed for pregnant, Keep away from children, Not allowed under 18, Not allowed for people with cardiovascular diseases, high blood pressure and lung diseases, not suitable for non-smokers	Acute toxic, Attention, Not allowed under 18, Not allowed for pregnant
G10	Germany	6	tobacco	Golden Blend	Nicotine contain, Keep away from children, Attention	Attention, Acute toxic,	Nicotine, Keep away from children, Attention	Attention, Acute toxic
UK4	United Kingdom	6	fruity	Strawberryade	No box	No box	Keep away from children, Nicotine	Attention, Acute toxic,

							contain, Harmful if swallowed	Not allowed under 18
P24	Poland	6	fruity	Green apple	No box	No box	Nicotine contain	Attention, Acute toxic
CZ1	Czech Republic	6	tobacco	Virginia Tobacco	Nicotine contain, Attention, Harmful if swallowed	No information	Attention, Acute toxic, Not allowed under 18, Not allowed for pregnant	No information
UK16	United Kingdom	6	fruity	Watermelon	Contains nicotine. Toxic if swallowed. Wash hands thoroughly after handling. Do not eat, drink or smoke when using this product. Keep out of reach of children	Danger, Acute toxic, Keep away from children, Nicotine contain	Contains Nicotine	Danger, Acute toxic, Keep away from children, Nicotine contain
CZ5	Czech Republic	6	tobacco	Tobacco	Nicotine contain, Attention, Harmful if swallowed	Danger, Acute toxic, Keep away from children, Nicotine contain	Attention, Acute toxic, Not allowed under 18, Not allowed for pregnant	Danger, Acute toxic, Keep away from children, Nicotine contain
UK20	United Kingdom	6	tobacco	Tobacco	No box	No box	Attention, Keep away from children, Nicotine contain	Attention, Acute toxic
C6	Croatia	6	sweet	Bluberry Cream	Acute toxic, Attention, Nicotine contain, Keep away from children	Acute toxic, Attention, Nicotine, Harmful after ingestion, Keep away from children	Acute toxic, Attention, Nicotine, Harmful after ingestion, Keep away from children	Acute toxic, Attention, Nicotine, Harmful after ingestion, Keep away from children
P20	Poland	6	fruity	Venom	No box	No box	Nicotine contain, Keep away from children, Toxic to the skin	Danger, acute toxic, Keep away from children, Not allowed under 18, Not allowed for pregnant
P21	Poland	6	fruit	Apple-Peach	No box	Danger, Nicotine contain, Toxic to the skin, Keep away from children	No box	Danger, acute toxic, Keep away from children, Not allowed under 18, Not allowed for pregnant
F1	France	4	sweet	Hip Toss	No box	No package	Attention, Keep away from children, Nicotine contain	Attention, Acute toxic
F2	France	4	sweet	NeckBreake r	No box	No box	Attention, Keep away from	Attention, Acute toxic

							children, Nicotine contain	
F3	France	4	sweet	Giant swing	Attention, Acute toxic, Nicotine, Keep away from children	Attention, Not allowed under 18, Not allowed for pregnant	Attention, Acute toxic, Nicotine contain, Keep away from children	Attention, Not allowed under 18, Not allowed for pregnant
P10	Poland	3	sweet	Coco	Danger, Nicotine, Acute toxic, Keep away from children	Danger, Acute toxic, Not allowed under 18, Not allowed for pregnant	Danger, Nicotine contain, Acute toxic, Keep away from children	Danger, Acute toxic
P11	Poland	3	unassigned	Energy Drink	Attention, Acute toxic, Keep away from children, Nicotine	Attention, Not allowed under 18, Not allowed for pregnant	Danger, Nicotine contain, Acute toxic, Keep away from children	Attention, Not allowed under 18, Not allowed for pregnant
P12	Poland	3	fruity	Sweet Cherry	Danger, Acute toxic, Keep away from children, Nicotine	Danger, Acute toxic	Danger, Nicotine contain, Acute toxic, Keep away from children	Danger, Acute toxic
P13	Poland	3	fruity	Strawberry-Mint	Acute toxic, Attention, Nicotine contain, Not allowed under 18, Keep away from children	Acute toxic, Attention	Acute toxic, Attention, Nicotine contain, Not allowed under 18, Keep away from children	Acute toxic, Attention
C1	Croatia	3	fruity	Cherry	Acute toxic, Attention, Nicotine contain, Keep away from children	Acute toxic, Attention, Nicotine, Harmful after ingestion, Keep away from children	Acute toxic, Attention, Nicotine, Harmful after ingestion, Keep away from children	Acute toxic, Attention, Nicotine, Harmful after ingestion, Keep away from children
P17	Poland	3	menthol	Sweet Mint	No package	No package	Toxic if swallowed, Nicotine contain, Keep away from children, Not allowed under 18	Attention, Acute toxic
G4	Germany	3	tobacco	American Blend Gold	Nicotine contain, Keep away from children, Attention	Attention, Acute toxic,	Nicotine, Keep away from children, Attention	Attention, Acute toxic
G9	Germany	3	tobacco	Golden Blend	No box	No box	Nicotine contain, Keep away from children, Not allowed under 18	No information
UK2	United Kingdom	3	fruity	Medusa	No box	No box	Harmful if swallowed, Not allowed under 18, Keep away from children, Nicotine contain	Attention, Acute toxic, Not allowed under 18, Keep away from children,
UK3	United Kingdom	3	sweet	Pink Ice	No box	No box	Nicotine contain, Keep away from children, Not allowed under 18, may contain cancerogenic	No information

							substances, Not allowed for pregnant, Not for people with cardiovascular diseases,	
UK5	United Kingdom	3	sweet	Guava Pop	No box	No box	Nicotine contain, Keep away from children, Harmful if swallowed	Attention, Acute toxic, Not allowed under 18, Not allowed for pregnant
UK6	United Kingdom	3	sweet	Honey Creme	No box	No box	Nicotine contain, Keep away from children, Harmful if swallowed	Attention, Acute toxic, Not allowed under 18, Not allowed for pregnant
UK7	United Kingdom	3	sweet	Birthday Cake	No box	No box	Nicotine contain, Keep away from children	Attention, Acute toxic, Not allowed under 18, Not allowed for pregnant
UK8	United Kingdom	3	sweet	Vanilla Custard Cream	No box	No box	Attention, Nicotine contain, Keep away from children, Not allowed under 18	Attention, Acute toxic
P19	Poland	3	sweet	Apple pie	No box	No box	Harmful if swallowed, Keep away from children, Nicotine contain, Not allowed under 18	Attention, Acute toxic, Not allowed under 18
UK10	United Kingdom	3	sweet	Strawberry Custard	No box	No box	Keep away from children, Harmful if swallowed, attention	Attention, Acute toxic, Keep away from children, Not allowed under 18, Not allowed for pregnant, Not allowed for consumption
UK12	United Kingdom	3	sweet	Kush Cake	Keep away from children, Attention	No information	Keep away from children, Not allowed under 18, Not allowed for pregnant	No information
P28	Poland	3	fruity	Strawberry with nectarine	Nicotine contain, Keep away from children, Toxic to the skin, Attention	No information	Attention, Acute toxic, Not allowed under 18	No information
P23	Poland	3	sweet	Apple Strudel	No package	No package	Nicotine contain, Keep away from	Attention, Acute toxic,

							children, Not allowed under 18, Not recommended for non-smokers	Not allowed under 18
P29	Poland	3	tobacco	Orient Tobacco	No box	No box	Nicotine Contain, Keep away from children	Harmful if swallowed, keep out of reach of children
UK14	United Kingdom	3	fruity	Cherry Bomb	Contains nicotine. Toxic if swallowed. Wash hands thoroughly after handling. Do not eat, drink or smoke when using this product. Keep out of reach of children	Danger, Acute toxic, Keep away from children, Nicotine contain	Contains Nicotine	Danger, Acute toxic, Keep away from children, Nicotine contain
UK15	United Kingdom	3	fruity	Watermelon	Contains nicotine. Toxic if swallowed. Wash hands thoroughly after handling. Do not eat, drink or smoke when using this product. Keep out of reach of children	Danger, Acute toxic, Keep away from children, Nicotine contain	Contains Nicotine	Danger, Acute toxic, Keep away from children, Nicotine contain
C7	Croatia	3	sweet	Blueberry Cream	Acute toxic, Attention, Nicotine contain, Keep away from children	Acute toxic, Attention, Nicotine, Harmful after ingestion, Keep away from children	Acute toxic, Attention, Nicotine, Harmful after ingestion, Keep away from children	Acute toxic, Attention, Nicotine, Harmful after ingestion, Keep away from children
UK9	United Kingdom	1.5	sweet	Vanilla Custard Cream	No box	No box	Nicotine contain, Keep away from children	Attention, Acute toxic, Not allowed under 18, Not allowed for pregnant
P22	Poland	1.5	fruity	Strawberry, Kiwi, Watermelon	No box	No box	Nicotine contain, Keep away from children, Not allowed under 18	No information
UK1	United Kingdom	1.5	sweet	Chocolate Cream Hazelnut	No box	No box	Nicotine contain, Keep away from children	No information
P14	Poland	0	fruity	Pineapple	Not allowed under 18, Not allowed for pregnant	Not allowed under 18, Keep away from children	Not allowed under 18, Keep away from children	Not allowed under 18, Keep away from children
P15	Poland	0	fruity	Orange	No box	No box	Not for consumption	Not allowed under 18, Keep away from children, Not allowed for pregnant,

C3	Croatia	0	tobacco	Tobacco	Acute toxic, Attention, Harmful after ingestion, Not for consumption, Keep away from children	No information	Harmful after ingestion, Keep away from children	Not for consumption Not allowed under 18, Keep away from children, Not allowed for pregnant, Not for consumption
G5	Germany	0	tobacco	American Blend Gold	Not allowed for pregnant, Keep away from children, Not allowed under 18, Not allowed for people with cardiovascular diseases, high blood pressure and lung diseases, not suitable for non-smokers, use can cause health problems	Not allowed under 18, Not allowed for pregnant	Not allowed for pregnant, Keep away from children, Not allowed under 18, Not allowed for people with cardiovascular diseases, high blood pressure and lung diseases, not suitable for non-smokers, use can cause health problems	Not allowed under 18, Not allowed for pregnant
G7	Germany	0	tobacco	Golden Blend	Keep away from children	No information	No information	No information
UK11	United Kingdom	0	fruity	Barracuda	No box	No box	Nicotine contain	Not allowed under 18, Attention, Acute toxic, Not allowed for pregnant, Not for consumption
P26	Poland	0	fruity	Gooseberry with Raspberry	Keep away from children	No information	Keep away from children, Not allowed under 18, Not allowed for pregnant	No information
P27	Poland	0	fruity	Wild Strawberry	Keep away from children	No information	Keep away from children, Not allowed under 18, Not allowed for pregnant	No information
UK13	United Kingdom	0	sweet	Lemon Sherbert	No box	No box	Propylene Glycol contain, Keep away from children	Harmful if swallowed, keep out of reach of children
UK19	United Kingdom	0	tobacco	Tobacco	Contains Propylene Glycol, Wash hands thoroughly after handling. Do not eat, drink or smoke when using this product.	Danger, Acute toxic, Keep away from children, Nicotine contain	No information	Danger, Acute toxic, Keep away from children, Nicotine contain

UK22	United Kingdom	0	fruity	Raspberry	Keep out of reach of children Contains Propylene Glycol, Wash hands thoroughly after handling. Do not eat, drink or smoke when using this product. Keep out of reach of children	Harmful if swallowed, keep out of reach of children	Keep away from children, Not allowed under 18, Not allowed for pregnant	Harmful if swallowed, keep out of reach of children
P33	Poland	0	fruity	Strawberry-lemon-orange	Contains Propylene Glycol, Wash hands thoroughly after handling. Do not eat, drink or smoke when using this product. Keep out of reach of children	Harmful if swallowed, keep out of reach of children	Keep away from children, Not allowed under 18, Not allowed for pregnant	No information
UK24	United Kingdom	0	fruity	Cherry	Contains Propylene Glycol, Wash hands thoroughly after handling. Do not eat, drink or smoke when using this product. Keep out of reach of children	Harmful if swallowed, keep out of reach of children	Keep away from children, Not allowed under 18, Not allowed for pregnant	Harmful if swallowed, keep out of reach of children
P34	Poland	0	sweet	Sweet Strawberry pie	No box	No box	Contains Propylene Glycol, Wash hands thoroughly after handling. Do not eat, drink or smoke when using this product. Keep out of reach of children	Harmful if swallowed, keep out of reach of children

Table S2. Results for e-liquids with nicotine level labeled as 0 or with no quantified nicotine level.

Sample code	Country	Flavour	Type of Flavour	Nicotine Concentration [mg/mL]	Experimental Nicotine Content [mg/mL]*	pH
P4	Poland	Montana tobacco	tobacco	6	ND	7.91
C3	Croatia	Tobacco	tobacco	0	0.05	3.89
G5	Germany	American Blend Gold	tobacco	0	ND	5.09
G7	Germany	Golden Blend	tobacco	0	ND	4.93
P14	Poland	Pineapple	fruity	0	ND	3.84
P15	Poland	Orange	fruity	0	ND	4.93
P26	Poland	Gooseberry with Raspberry	fruity	0	0.02	3.84
P27	Poland	Wild Strawberry	fruity	0	ND	4.40
P33	Poland	Strawberry-lemon-orange	fruity	0	ND	3.38
P34	Poland	Sweet Strawberry pie	sweet	0	ND	2.98
UK11	United Kingdom	Barracuda	fruity	0	ND	4.54
UK13	United Kingdom	Lemon Sherbert	sweet	0	ND	3.80
UK19	United Kingdom	Tobacco	tobacco	0	ND	4.25

UK22	United Kingdom	Raspberry	fruity	0	ND	3.97
UK24	United Kingdom	Cherry	fruity	0	ND	4.71

Note: * limit of quantification: 0.02 mg/mL; limit of detection: 0.007 mg/mL; ND: not detected.
